# Prognostic value of visual IMPeTUs criteria and metabolic tumor burden at baseline [^18^F]FDG PET/CT in patients with newly diagnosed multiple myeloma

**DOI:** 10.1186/s13550-024-01113-6

**Published:** 2024-05-28

**Authors:** Silvano Marchiori, François Cousin, Iraklis Papadopoulos, Claire Bernard, Marie Thys, Bernard De Prijck, Michelle Pirotte, Anne-Françoise Donneau, Roland Hustinx, Jo Caers, Nadia Withofs

**Affiliations:** 1 Division of Nuclear Medicine and Oncological Imaging, Department of Medical Physics, CHU of Liege, Liège, Belgium; 2 Biostatistics and Research Methods Center (B-STAT), University of Liege, Liège, Belgium; 3 Medico-Economic Information Department / Data Analysis, CHU of Liège, Liège, Belgium; 4 Department of Hematology, CHU de Liège, Liège, Belgium; 5 GIGA-CRC in Vivo Imaging, University of Liege, Liège, Belgium; 6 Laboratory of Hematology, GIGA I3, University of Liège, Liège, Belgium

**Keywords:** Myeloma, PET, FDG, Imaging, MTV

## Abstract

**Background:**

2-[^18^F]fluoro-2-deoxy-D-glucose ([^18^F]FDG) positron emission tomography combined with low-dose computed tomography (PET/CT) can be used at diagnosis to identify myeloma-defining events and also provides prognostic factors. The aim of this study was to assess the prognostic significance of baseline [^18^F]FDG PET/CT visual IMPeTUs (Italian myeloma criteria for PET Use)-based parameters and/or total metabolic tumor volume (TMTV) in a single-center population of patients with newly diagnosed multiple myeloma (NDMM) eligible for transplantation.

**Methods:**

Patients with MM who underwent a baseline [^18^F]FDG PET/CT were retrospectively selected from a large internal database of the University Hospital of Liege (Liege, Belgium). Initially, all PET/CT images were visually analyzed using IMPeTUs criteria, followed by delineation of TMTV using a semi-automatic lesion delineation workflow, including [^18^F]FDG-positive MM focal lesions (FL) with an absolute SUV threshold set at 4.0. In a first step, to ensure PET/CT scans accurate reporting, the agreement between two nuclear medicine physicians with distinct experience was assessed. In the second step, univariable and multivariable analyses were conducted to determine the prognostic significance of [^18^F]FDG PET/CT parameters on progression free survival (PFS) and overall survival (OS), respectively.

**Results:**

A total of 40 patients with NDMM were included in the study. The observers agreement in the analysis [18F]FDG PET/CT images was substantial for the presence of spine FL, extra spine FL, at least one fracture and paramedullary disease (Cohen’s kappa 0.79, 0.87, 0.75 and 0.64, respectively). For the presence of skull FL and extramedullary disease the agreement was moderate (Cohen’s kappa 0.56 and 0.53, respectively). Among [^18^F]FDG PET/CT parameters, a high number of delineated volumes of interest (VOI) using the SUV4.0 threshold was the only independent prognostic factor associated with PFS [HR (95% CI): 1.03 (1.004–1.05), *P* = 0.019] while a high number of FL (*n* > 10; F group 4) was the only independent prognostic factor associated with OS [HR (95% CI): 19.10 (1.90–191.95), *P* = 0.01].

**Conclusion:**

Our work confirms the reproducibility IMPeTUs criteria. Furthermore, it demonstrates that a high number of FL (*n* > 10; IMPeTUs F group 4), reflecting a high [^18^F]FDG-avid tumor burden, is an independent prognostic factor for OS. The prognostic value of the TMTV delineated using a SUV4.0 threshold was not significant. Nevertheless, the count of delineated [^18^F]FDG-avid lesions VOI using a SUV4.0 threshold was an independent prognostic factor for PFS.

**Supplementary Information:**

The online version contains supplementary material available at 10.1186/s13550-024-01113-6.

## Introduction

Multiple myeloma (MM) is the second most common hematological malignancy with higher incidence in high-income countries, particularly in Western Europe, North America and Australasia [1]. MM is characterized by a clonal plasma cell proliferative disorder, demonstrating significant clonal heterogeneity and consequently highly variable clinical outcomes [2]. Induction therapy followed by autologous stem-cell transplantation (ASCT) and novel therapeutic agents have improved the prognosis of MM patients, elevating the median survival from 2–3 years to 7–15 over the past two decades [3, 4]. In patients with newly diagnosed MM (NDMM), the revised International Staging System (R-ISS) is used as a simple prognostic tool to categorize patients into three stage groups with distinct survival rate [5, 6]. Patients age, performance status, comorbidities, and the depth of response to therapy are also well-known prognostic factors [2, 7]. Additional biomarkers are still needed to identify high-risk patients for whom therapy could maximize response, as well as standard-risk patients for whom unnecessary treatment-related toxicity can be minimized.

2-[^18^F]fluoro-2-deoxy-D-glucose ([^18^F]FDG) positron emission tomography combined with low-dose computed tomography (PET/CT can be employed at the time of diagnosis to detect myeloma-defining events; it also provides prognostic insights [8–10]. In contrast to chromosomal abnormalities assessed in a single bone marrow sample, total body [^18^F]FDG PET/CT scan may help to capture the intra-individual spatial heterogeneity of the disease, identifying high-risk lesions [11]. The presence of extramedullary disease (EMD) and/or the presence of [^18^F]FDG avid focal lesions (FL) with standardized uptake value (SUV) > 4.2 independently and adversely impact progression free survival (PFS) and overall survival (OS) [12–15]. The visual IMPeTUs (Italian myeloma criteria for PET USe) criteria developed by Nanni et al. have successfully contributed to the standardization of [^18^F]FDG PET/CT reports of NDMM patients in clinical trials [16–18]. The current endeavor focuses on identifying and selecting parameters to simplify these criteria, retaining only those with prognostic value and implications for therapeutic decision-making*.* Deng et al. showed that bone marrow (BM) Deauville-scale (DS) score ≥ 4 was independently associated with OS [hazard ratio (HR) 3.49; 95% confidence interval (CI), 1.12–10.85, *P* = 0.031] [19]. Sachpekidis et al*.* [15, 20] identified that the number of bone [^18^F]FDG-avid FL was associated with significantly shorter PFS in the multivariate analysis. Both confirmed EMD being an independent prognostic factor [15, 19]. More recently, it has been revealed that the presence of paramedullary disease (PMD) in the patients of the CASSIOPET trial, was independently associated with PFS [HR 3.16; 95% CI, 1.60–6.28, *P* = 0.001] [14, 21]. Research groups also illustrated that baseline total metabolic tumor volume (TMTV) was independently associated with PFS and OS [20, 22–28]. The threshold mostly used by research groups to delineate the TMTV was SUV2.5 [20, 22–28]. Using a random survival forest approach including radiomic features derived from [^18^F]FDG PET/CT of transplant-eligible NDMM patients of two prospective independent randomized phase III IFM/DFCI2009 and EMN02/HO95 trials, Jamet et al. highlighted that the received treatment, the presence of anemia, and high diffuse BM maximum SUV (SUV_max_) were the three most predictive variables of PFS [29].

The aim of this study was to evaluate the prognostic significance of baseline [^18^F]FDG PET/CT visual IMPeTUs-based parameters and/or TMTV in a single-center population of NDMM.

## Materials and methods

### Study population

The approval was obtained from the institutional ethics committee (Comité d'Éthique Hospitalo-Facultaire Universitaire de Liège; reference 2023/9). No written informed consent was obtained due to the retrospective design of the study. Patients diagnosed with plasma-cell disorders who had undergone a PET/CT scan were retrospectively extracted from a large internal database of the University Hospital of Liege (Liege, Belgium), covering the period from 2012 to 2021. A subsequent selection was performed with the following eligibility criteria: NDMM without prior treatment, active MM requiring induction therapy and eligible for transplant; age ≤ 70 year (usual cutoff age for establishing transplant eligibility); availability of baseline [^18^F]FDG PET/CT within a maximum interval of three months the MM diagnosis requiring therapy. Exclusion criteria encompassed cases where immediate treatment initiation did not occur, follow-up information was unavailable, and cases where the [^18^F]FDG PET/CT did not meet the quality criteria, such as glycemia > 150 mg/dL at the time of [^18^F]FDG injection.

### [^18^F]FDG PET/CT analyses

[^18^F]FDG PET/CT acquisition parameters are presented in Supplemental Data.

[^18^F]FDG PET/CT images were independently analyzed by an experienced nuclear medicine physician (18-year experience) and a trainee in nuclear medicine (2-year experience in nuclear medicine) using MIM Software version 7.0.5 (LesionID® tool; MIM Software Inc.). To ensure PET/CT scans accurate reporting, the agreement between the two nuclear medicine physicians was assessed in the qualitative and quantitative description of PET/CT images. In a second step, consensus between the two nuclear medicine physicians was obtained for the IMPeTUs variables that were then used in the Cox proportional hazards models. The consensus results were also compared with the clinical report provided by the third physician involved in the PET/CT report as part of routine clinical practice. The CT part of the PET/CT was further analyzed by a radiologist experienced in bone imaging and MM. All PET/CT images were first visually analyzed using IMPeTUs criteria [16, 17]. The following parameters were reported: The presence of positive FL with [^18^F]FDG uptake with or without underlying osteolytic lesion in CT images; the presence of at least one osteolytic lesion of 5 mm or more in size with or without [^18^F]FDG uptake; the number of FL (F) and osteolytic lesions (L), respectively, merged into the following groups × 1: no lesion; × 2: 1–3 lesions; × 3: 4–10 lesions and × 4: > 10 lesions; the site of MM lesions (skull, spinal and/or extraspinal); the intensity of [^18^F]FDG uptake in the BM and the hottest FL, respectively, quantified according to the five-point Deauville scale and SUV_max_; the presence and site of EMD and/or PMD; the presence of fractures; the presence of a diffuse pattern in CT images (innumerable osteolytic lesions distributed diffusely throughout the axial skeleton).

The liver SUV_max_ and SUV_mean_ were measured using a 30-mm diameter volume of interest (VOI). Whole-body [^18^F]FDG-positive tumor volume delineation was performed using a semi-automatic lesion delineation workflow of MIM Software version 7.0.5 (MIM Software Inc.). A rectangular volume of interest including the whole body was manually selected. Then, a fully automated preselection of [^18^F]FDG-positive MM FL was applied to delineate lesions with an absolute SUV threshold set at 4.0. No minimum or maximum volume threshold was applied. The observer used a clearing option to manually remove areas of physiological uptake/activity, e.g., heart, brain, bladder. The software then provided the SUV_max_, SUV_mean_, the number of VOI delineated using the SUV4.0 threshold, the TMTV and the total lesion glycolysis (TLG). For patients with no delineated volume (patients with no FL or FL with SUV_max_ < 4), the SUV_max_ was estimated manually by drawing a VOI in the hottest FL. Semi-quantitative values of the experienced nuclear medicine physician were used in the Cox proportional hazards models.

### Outcomes

The primary endpoint was to test the association between baseline [^18^F]FDG PET/CT IMPeTUs criteria and semi-quantitative parameters (TMTV and SUVs) and PFS and OS, respectively. The PFS was defined as the duration from the date of diagnosis of active MM requiring treatment until either the date when progression criteria were met or the date of death, whichever occurred earlier. Patients without documented progression after diagnosis and without a recorded death date were censored for PFS at their last contact date. The OS was defined as the time from the date of diagnosis of active MM requiring treatment to the time of death from any cause; patients who were still alive at the last follow-up or lost to follow-up were considered as censored.

### Statistical analyses

Patient characteristics and PET parameters were summarized using median and interquartile range (P25-P75). The normality of the distribution of quantitative variables was assessed through numerical comparison of mean and median values, graphical representations with histograms and quantile–quantile plots as well as the Shapiro–Wilk normality test. Categorical covariates were reported as absolute and relative frequencies. The agreement between variables measured by both observers was evaluated using Cohen’s Kappa coefficient for qualitative variables. In the case of quantitative variables, the agreement was assessed using intra-class correlation (ICC). Both, Cohen’s Kappa and ICC are presented along with their corresponding 95% Confidence Intervals (CIs). The values of Cohen’s kappa and ICC ranged from 0 to 1, and guideline for interpreting the degree of agreement was as follows: total disagreement ≤ 0.01, slight agreement = 0.01–0.20, fair agreement = 0.21–0.40, moderate agreement = 0.41–0.60, substantial agreement = 0.61–0.80, and almost perfect agreement = 0.81–1.00. Kaplan–Meier plots were employed to visualize PFS and OS curves. Hazard ratios (HRs) with corresponding 95% CI were derived to access potential clinical and PET/CT risk factors for time to death and time to progression after diagnosis using Cox proportional hazards models (Cox-ph). Univariable and multivariable analyses were conducted for both PFS and OS. Results were considered significant at the 5% level of significance (*P* < 0.05). Analyses were carried out on the maximum of data availability; the missing values were not replaced. Calculations, visualization, and modelling were performed using R programming-version 4.2.2.

## Results

### Patients

**Fig. 1:**
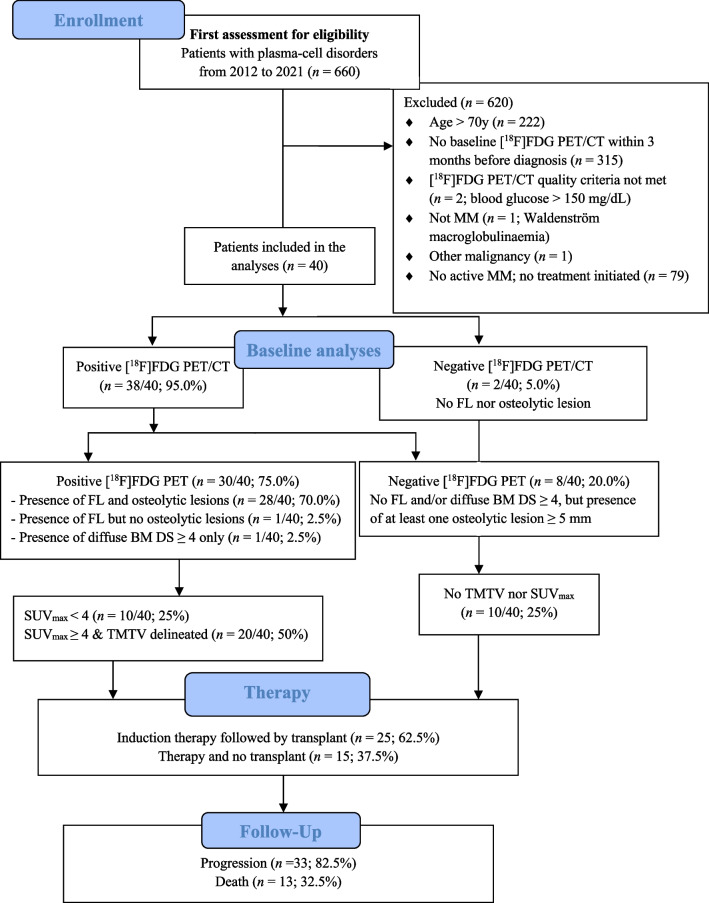
Consort flow diagram. Abbreviations: [^18^F]FDG, 2-[^18^F]fluoro-2-deoxy-D-glucose;BM, bone marrow; DS, Deauville scale; FL, focal lesion; MM, multiple myeloma; PET/CT, positron emission tomography/computed tomography; SUV_max_, maximum standardized uptake value; TLG, total lesion glycolysis; TMTV, total metabolic tumor volume; VOI, volume of interest

The consort flow diagram is presented in the Fig. 1. A total of 660 patients with plasma-cell disorders, and for whom a PET/CT was performed, were selected. A total of 620 patients did not meet the eligibility criteria and were excluded for various reasons: age > 70 year (*n* = 222); absence of baseline [^18^F]FDG PET/CT within three months before MM diagnosis (*n* = 315); [^18^F]FDG PET/CT not meeting the quality criteria (*n* = 2; glycemia > 150 mg/dL); no active MM and no treatment initiated (*n* = 79), presence of other malignancy (*n* = 1); and misdiagnosis of MM (*n* = 1; Waldenström macroglobulinaemia).

A total of 40 patients with NDMM were included in the study. The first MM diagnosis was made on January 21, 2011, and the last on March 9, 2021. Monoclonal gammopathy of undetermined significance was present in 6/40 (15.0%) patients before the active MM diagnosis. Patient characteristics are presented in Table 1. Disease stage ISS I (*n* = 21/40; 52.5%) and R-ISS II (*n* = 21/40; 52.5%) were observed in majority of the patients. Transplant was not performed after induction in 15/40 (37.5%) patients due to progression (n = 8/15), comorbidities (n = 6/15) and significant residual disease (n = 1/15). Transplant was performed in 25/40 (62.5%) patients, with single ASCT performed in 18/25 patients, tandem ASCT performed in 7/25 patients and allogeneic SCT performed in 1/25 patients (Table 1).

**Table 1:** Patient characteristics

Characteristic	Value n = 40 (100%)	Specified if missing, n (%)
Median (P25-P75) age	63.2 (55.8–68.1) years	
Sex: Male	22 (55.0%)	
IgG MM IgA MM Light chain MM	16 (40.0%) 13 (32.5%) 9 (22.5%)	
Non-secretory MM	2 (5.0%)	
Median (P25-P75) hemoglobin	10.50 (10.32–12.93) g/dL	2 (5.0%)
ISS stage I	21 (52.5%)	
ISS stage II	10 (25.0%)	
ISS stage III	6 (15.0%)	
R-ISS stage I	11 (27.5%)	
R-ISS stage II	21 (52.5%)	
R-ISS stage III	5 (12.5%)	
R-ISS and ISS stage missing		3 (7.5%)
Median (P25-P75) serum β2-microglobulin	3.30 (2.27–4.37) mg/L	7 (17.5%)
Serum β2-microglobulin > 5.5 mg/L	5 (12.5%)	
Serum albumin < 3.5 g/dL	8 (20.0%)	
Median (P25-P75) LDH	190 (146–220) U/L	2 (5.0%)
Serum LDH > the upper limit of normal (220 U/L)	13 (32.5%)	1 (2.5%)
Presence of high-risk cytogenetic abnormalities^†^	12 (30.0%)	
Mean (SD) serum M protein IgG/IgA	26.7 (17.0) g/L	12 (30.0%)
Median (P25-P75) serum FLC (kappa/lambda) ratio	2.20 (0.0400–65.2)	5 (12.5%)
Median (P25-P75) serum creatinine	0.940 (0.710–1.26) mg/dL	3 (7.5%)
Median (P25-P75) serum calcium	2.44 (2.36–2.52) mmol/L	3 (7.5%)
Median (P25-P75) BMPC percentage	35.0 (19.0–67.0) %	2 (5.0%)
First-line treatment received:		
Bortezomib, thalidomide & dexamethasone (VTd)	25 (62.5%)	
Bortezomib, cyclophosphamide & dexamethasone (VCd)	3 (7.5%)	
Lenalidomide, bortezomib & dexamethasone (VRd)	2 (5.0%)	
Bortezomib, melphalan & prednisone (VMP)	2 (5.0%)	
Bortezomib & dexamethasone	1 (2.5%)	
Daratumumab, bortezomib & dexamethasone (DVd)	1 (2.5%)	
Lenalidomide & dexamethasone	3 (7.5%)	
Daratumumab, lenalidomide & dexamethasone (DRd)	2 (5.0%)	
Lenalidomide and methylprednisolone	1 (2.5%)	
Autologous stem cell transplant Allogeneic stem cell transplant No transplant	24 (60%) 1 (2.5%) 15 (37.5%)	

Data are presented as numbers (%), unless otherwise specified

BMPC, clonal bone marrow plasma cell; FLC, free light chain; ISS, International Staging System; LDH, lactate dehydrogenase; R-ISS, revised-ISS

^†^High risk cytogenetics included t(4;14), t(14;16), or del(17p)

### [^18^F]FDG PET/CT results

The PET/CT interpretation results are illustrated in the consort flow diagram (Fig. 1) and in Suppl. Table 1. [^18^F]FDG PET/CT was negative (no FL lesion nor osteolytic lesion and no BM uptake with DS ≥ 4) in only 2/40 (5.0%) patients. Eight patients out of 40 (20.0%) had at least one osteolytic lesion measuring 5 mm or more (L group > 1) but no FL (F group 1) and no diffuse BM [^18^F]FDG uptake with DS ≥ 4. [^18^F]FDG PET/CT showed the presence of both FL (F group > 1) and osteolytic lesions (L group > 1) in 28/40 (70.0%) patients. Two out of 40 (5.0%) patients had no osteolytic lesion but presented at least one FL with [^18^F]FDG uptake (*n* = 1; confirmed by magnetic resonance imaging) or diffuse BM [^18^F]FDG uptake with DS ≥ 4 (*n* = 1). [^18^F]FDG PET showed FL with DS ≥ 4 in 19/40 (47.5%) patients and/or diffuse BM [^18^F]FDG uptake with DS ≥ 4 in 8/40 (20.0%) patients. Paramedullary disease was identified in 12 (30.0%) patients, out of which a DS ≥ 4 was recorded in 9/40 (22.5%) patients; PMD was detected with CT only in one patient (no [^18^F]FDG uptake; F group 1). Two (5.0%) patients showed at least one EMD, with one patient presenting with a paravertebral pleural mass (DS 3; no biopsy performed) and another patient exhibiting an epiglottic lesion and one locoregional lymph node (DS 4; no biopsy performed; these lesions disappeared at the post-induction [^18^F]FDG PET/CT).

**Fig. 2:**
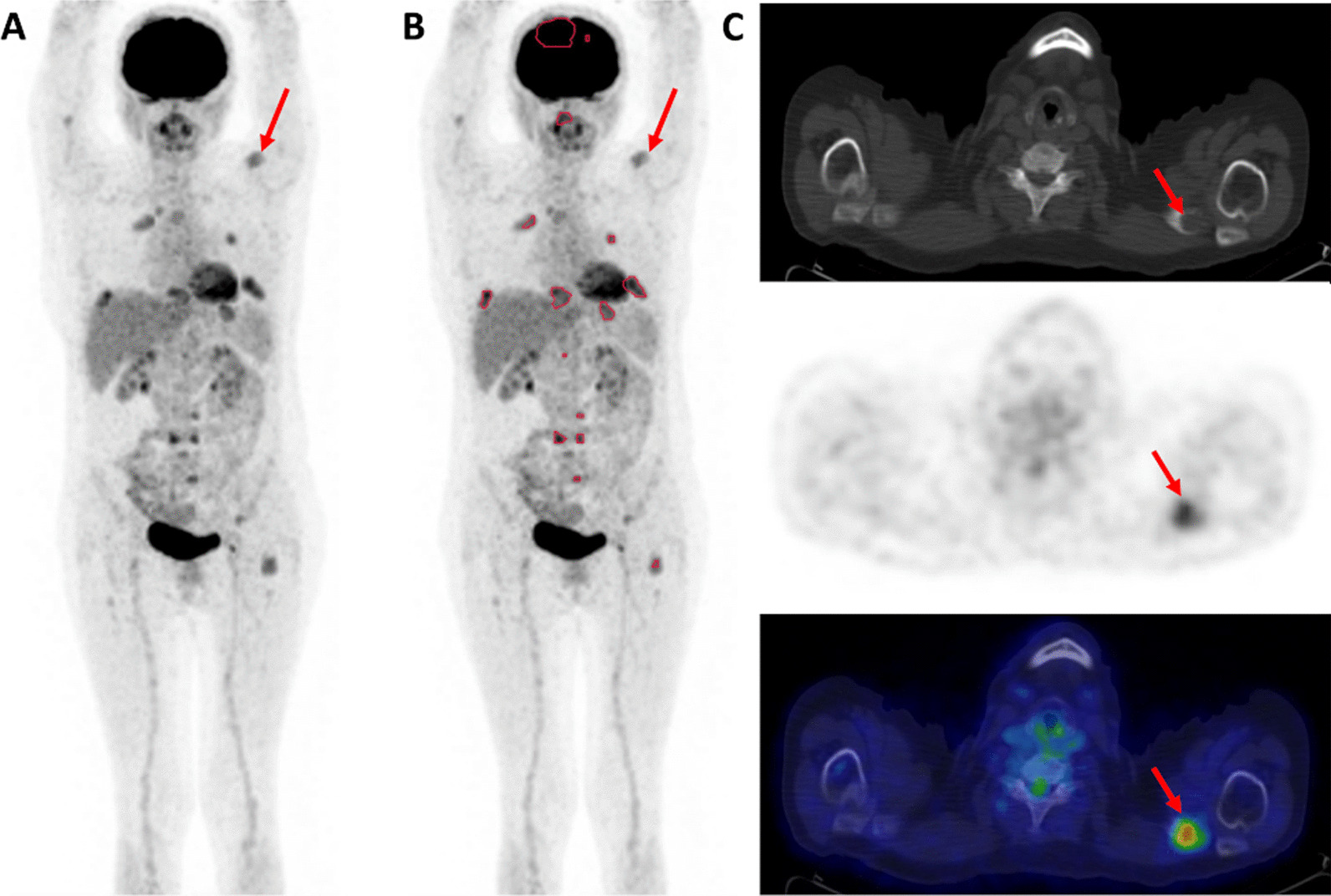
[^18^F]FDG PET images [**A** & **B**: maximum intensity projection (MIP); C: PET/CT axial slices; SUV scale 0–5] of a patient with multiple FL (F group 4; *n* = 16 delineated VOI). This 65-year-old patient was diagnosed with IgG kappa MM (ISS and R-ISS stage II; serum M-protein 54.3 g/L; free light-chain Kappa 79.17 mg/L, abnormal free light-chain ratio Kappa/Lambda: 34.88; bone marrow plasma cell infiltration (BMPC): 73%; hemoglobin 11.0 g/dL). Images show the TMTV (70.91 mL) delineated using the SUV4.0 threshold **B**. The figure illustrates the underestimation of tumor burden using SUV4.0 threshold; FL with SUV_max_ < 4.0 were not delineated, such as the osteolytic lesion of the left clavicle, pointed out by the red arrows, with [^18^F]FDG uptake but SUV_max_ 3.9. Note that this patient presented two delineated skull FLs, one of which was large with paramedullary extension. The patient underwent induction therapy with bortezomib, thalidomide and dexamethasone followed by ASCT. Time to progression was 22.8 months and time to death was 4.9 years

The median (range) liver SUV_max_ and SUV_mean_ were 3.1 (2.5–4.2) and 2.2 (1.7–2.8), respectively. Using the SUV4.0 threshold, it was possible to delineate the TMTV and extract TLG and SUV_mean_ in 20/40 (50.0%) patients who had at least one MM FL and/or diffuse BM uptake with SUV_max_ ≥ 4 (Fig. 2). No SUV_max_ was available in the 8/40 (20.0%) patients with no FL.

The agreement between the 2 nuclear medicine physicians in [^18^F]FDG PET/CT images analyses is presented in Table 2. Almost perfect agreement (ICC value > 0.90) was observed for all semi-quantitative parameters (TMTV, TLG, SUVs and the number of delineated VOI), for the group number of FL, the hottest bone FL DS and PMD DS. Substantial agreement (Cohen’s kappa or ICC value > 0.60) was observed for the BM DS, the group number of osteolytic lesions, the presence of fracture and PMD. The agreement was moderate (Cohen’s kappa < 0.60) for the presence of skull FL and EMD.

**Table 2:** Agreement between the 2 nuclear medicine physicians in [^18^F]FDG PET/CT images analyses

[^18^F]FDG PET/CT parameter	*n* = 40 (100%) if not specified	Cohen’s Kappa (95% CI)	ICC (95% CI)
BM DS			0.66 (0.44–0.80)
Presence of skull FL	33 (82.5)^††^	0.56 (0.31–0.80)	
Presence of spinal FL		0.79 (0.59–0.98)	
Presence of extraspinal FL		0.87 (0.71–1.00)	
Number of bone FL by groups			0.97 (0.94–0.98)
Hottest bone FL DS	32 (80.0)^†^		0.92 (0.83–0.96)
Number of CT osteolytic lesions by groups			0.71 (0.49–0.84)
Presence of at least one fracture in CT images		0.75 (0.56–0.95)	
Presence of PMD		0.64 (0.36–0.91)	
PMD DS	12 (30.0)		0.94 (0.69–0.99)
Presence of EMD		0.53 (0.27–0.72)	
TMTV	20 (50.0)		1.00 (1.00–1.00)
TLG	20 (50.0)		1.00 (1.00–1.00)
SUV_max_	32 (80.0)^†^		0.99 (0.97–1.00)
SUV_mean_	20 (50.0)		0.99 (0.98–1.00)
Number of volume of interest per patient			0.98 (0.96–0.99)

BM, bone marrow; CI, confidence interval; CT, computed tomography; DS, Deauville scale; EMD, extramedullary disease; FL, focal lesions; ICC: intraclass correlation coefficient; PMD, paramedullary disease; SUV, standardized uptake value; TLG, total lesion glycolysis; TMTV, total metabolic tumor volume

^†^ No focal lesion in 8 patients. ^††^Absence of skull FL but the skull vault was not included in the field of view

### Outcome results

**Fig. 3:**
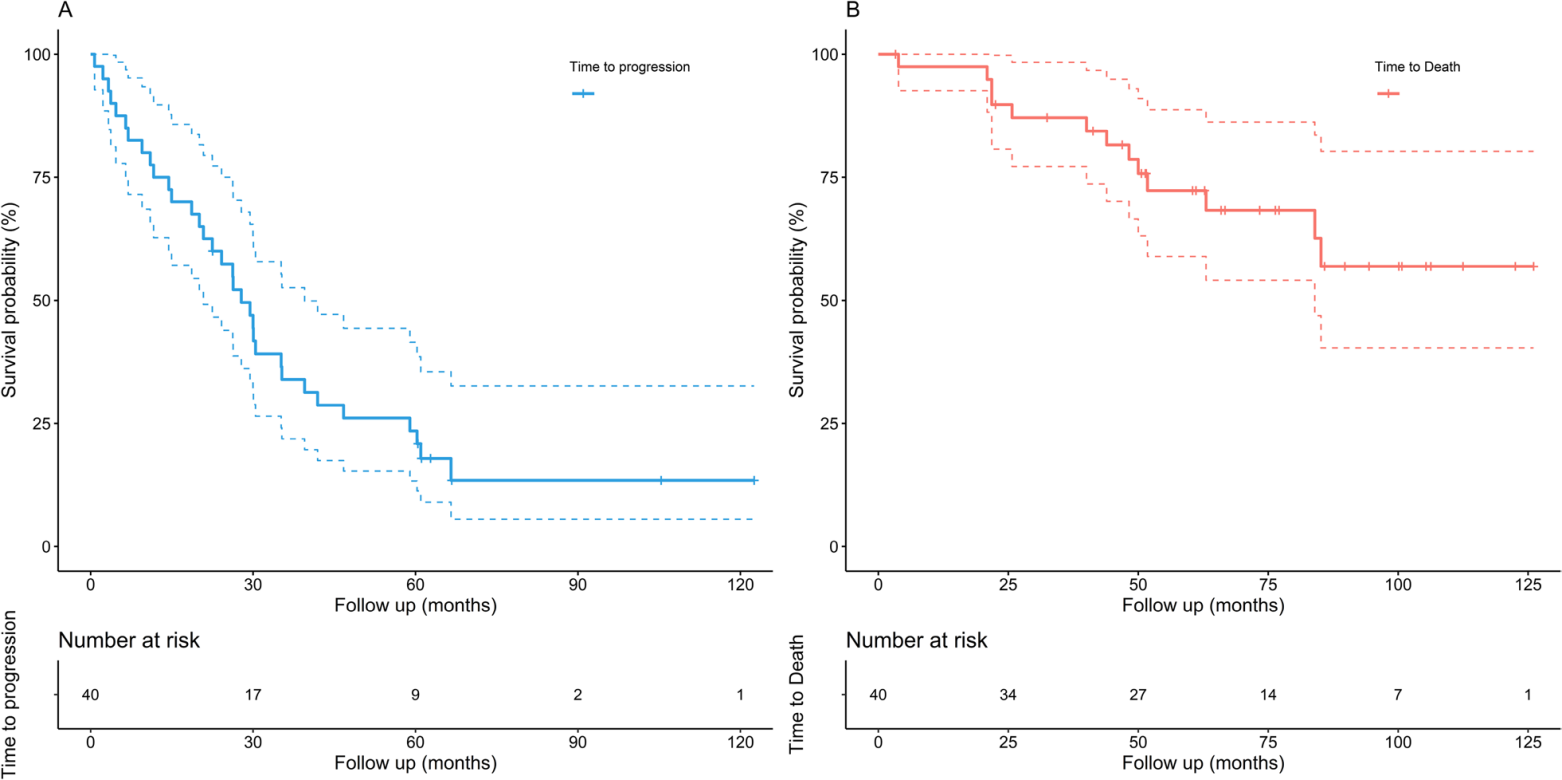
Kaplan–Meier curve for PFS **A** and OS **B**

Figure 3 presents the Kaplan–Meier curve for PFS and OS. Median (P25-P75) follow-up for OS time was 60.8 (43.3–85.3) months. The 3-year rate of PFS was 33.3% (95% CI 21.9–52.6). The 3-year rate of OS was 87.1% (95% CI 77.2–98.3). During the entire follow-up period, 33/40 (82.5%) patients progressed (PFS event) and 13/40 (32.5%) patients died (OS event).

The univariable and multivariable analyses evaluating the impact of clinical and [^18^F]FDG PET/CT variables on PFS and OS are presented in Table 3. In our population, two variables were significantly and independently associated with PFS: Performing transplant [HR (95% CI): 0.33 (0.12–0.87), *P* = 0.025] and the count of delineated VOI using the SUV4.0 threshold [HR (95% CI): 1.03 (1.004–1.05), *P* = 0.019]. The two variables significantly and independently associated with OS were the presence of high-risk cytogenetic abnormalities, [t(4;14), t(14;16) or del(17p); HR (95% CI): 14.97 (2.85–78.51), *P* = 0.001] and a high number of FL [*n* > 10; F group 4; HR (95% CI): 19.10 (1.90–191.95), *P* = 0.01].

**Table 3:** Multivariable cox regression analysis of clinical and baseline [^18^F]FDG PET/CT parameters predicting for prolonged PFS and OS^1, 2^

		PFS				OS			
		Univariable analysis		Multivariable analysis		Univariable analysis		Multivariable analysis^3^	
Parameter	Categories	HR (95% CI)	*P*-value	HR (95% CI)	*P*-value	HR (95% CI)	*P*-value	HR (95% CI)	*P*-value
Presence of high-risk cytogenetic abnormalities	Yes (Ref: No)	2.14 (1.02–4.49)	0.043			8.12 (2.25–29.27)	0.001	14.97 (2.85–78.51)	0.001
Transplant	Yes (Ref: No)	0.48 (0.23–0.98)	0.043	0.33 (0.12–0.87)	0.025	1.07 (0.33–3.47)	0.915		
BM DS	DS ≥ 4 (Ref: DS < 4)	2.46 (1.04–5.83)	0.040	1.29 (0.22- 7.52)	0.779	2.47 (0.65–9.39)	0.184		
Hottest bone FL DS	DS 3 (Ref: DS 2)	1.16 (0.29–4.72)	0.837			0.15 (0.02–1.03)	0.053		
	DS 4 (Ref: DS 2)	0.36 (0.07–1.19)	0.223			0.09 (0.009–0.88)	0.038		
	DS 5 (Ref: DS 2)	1.07 (0.29–3.91)	0.921			0.40 (0.10–1.70)	0.217		
Hottest bone FL DS ≥ 4	Yes (Ref: No)	0.70 (0.30–1.66)	0.420			0.82 (0.26–2.60)	0.733		
F group	F group 2 (Ref: F1)	0.78 (0.34–1.82)	0.569			5.38 (0.65–44.80)	0.119	3.74 (0.38–36.81)	0.257
	F group 3 (Ref: F1)	0.62 (0.193–1.983)	0.419			2.22 (0.14–36.06)	0.574	2.25 (0.12–41.48)	0.584
	F group 4 (Ref: F1)	1.26 (0.47–3.38)	0.641			12.92 (1.48–113.11)	0.020	19.10 (1.90–191.95)	0.010
Number of delineated VOI		1.01 (1.003–1.02)	0.008	1.03 (1.004- 1.05)	0.019	1.01 (1–1.02)	0.051		

VOI, volume of interest

^1^An interaction term between the significant parameters in the multivariate analyses was included in the model (*P* > 0.05)

^2^Results are adjusted for the following parameters tested in the univariate analyses: Age; gender; hemoglobin; ISS stage; R-ISS stage; serum β2-microglobulin; serum albumin; serum lactate dehydrogenase (LDH); serum calcium; [^18^F]FDG PET scan status defined positive in case of F group > 1 (at least one FL) and/or diffuse BM uptake with DS ≥ 4; the PET scan status defined negative in case of F group 1 (no FL) and no diffuse BM uptake with DS ≥ 4; F group 2 and 3; number of bone FL > 3; presence of at least one fracture; presence of PMD; presence of PMD with DS ≥ 4; presence of EMD; TMTV; TLG; hottest FL SUV_max_; hottest SUV_max_ > 4.2; SUV_mean_; more than 3 delineated VOI and CT diffuse pattern (*P* > 0.05)

^3^The multivariate analysis for OS is constrained by the low number of deaths (13 out of 40; 32.5%) observed during the follow-up period

## Discussion

The IMPeTUs criteria, as demonstrated by Nanni et al., proved to be highly reproducible among expert nuclear medicine physicians [17]. This work confirms the utility of IMPeTUs criteria and semi-quantitative parameters in generating consistent reports for [^18^F]FDG PET/CT scans, even when interpreted by less experienced residents in nuclear medicine. This emphasizes the benefit of using IMPeTUs descriptive criteria to standardize PET interpretation not only in clinical trials but also in routine clinical practice. The concordance in interpreting and quantifying [^18^F]FDG PET/CT scans was substantial or almost perfect for the majority of parameters, with the exception of the presence of skull FL and EMD, for which the agreement was moderate (Cohen’s kappa < 0.60). The low prevalence of EMD (*n* = 2/40; 5.0%) may account for this lack of agreement. For skull lesions, anatomic variants such as arachnoid granulations and benign lesions can mimic osteolytic MM lesions in CT images (false positive results) while FL can be missed due to high brain [^18^F]FDG uptake (false negative results) [30, 31].

Baseline [^18^F]FDG PET/CT was positive in the majority of newly diagnosed transplant-eligible MM patients (*n* = 38/40; 95.0%) included in this study. Half of the patients (*n* = 20/40) had diffuse BM uptake and/or FL with DS ≥ 4. Therefore, conducting a baseline [^18^F]FDG PET/CT scan is advisable when planning a post-treatment PET evaluation [32]. These 20 patients with MM involvement and DS ≥ 4 were those for whom the TMTV was delineable using the SUV4.0 threshold.

**Fig. 4:**
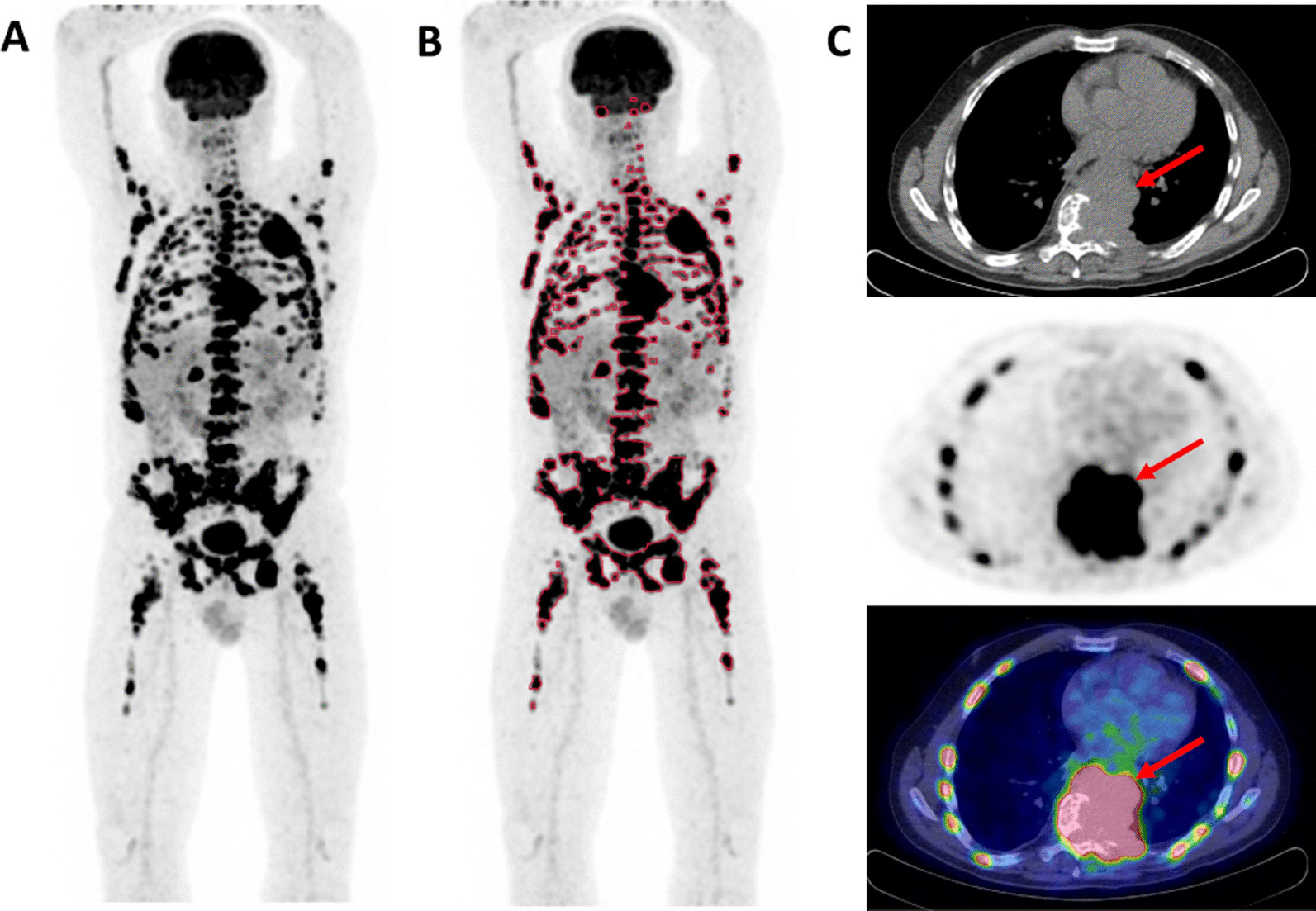
[^18^F]FDG PET/CT images (**A**: PET MIP; B: PET MIP with SUV4.0 threshold TMTV & C: low dose CT, PET and fused PET/CT; SUV scale 0–5) of a 49-year-old patient with newly diagnosed IgA kappa MM (ISS and R-ISS stage III; serum M-protein 7.3 g/L; free light-chain Kappa 4187 mg/L, abnormal free light-chain ratio Kappa/Lambda: 1495; hypercalcemia; bone marrow plasma cell infiltration: 25%; presence of t(4;14); hemoglobin 11.3 g/dL). The PET images show a high diffuse BM [^18^F]FDG uptake with a DS 5 and multiple FL (F group 4; 198 delineated VOI; TMTV 1369,7 ml) with DS 5. The red arrows in PET/CT images **C** point PMD with spinal cord compression confirmed by magnetic resonance imaging. The patient underwent involved-field radiation therapy of the spinal lesion and induction therapy with daratumumab, lenalidomide and dexamethasone followed by ASCT. Time to progression was 6.9 months and time to death was 21.8 months

According to the latest International Myeloma Working Group consensus recommendations, diffuse [^18^F]FDG uptake in BM is not a myeloma-defining event to avoid false diffuse BM pattern related to reactive BM [8–10]. The grading of diffuse BM uptake using the 5-point scale Deauville score is among the IMPeTUs criteria [16–18]. In our population, 8/40 (20.0%) patients presented with diffuse BM DS ≥ 4, with BM plasma cell infiltration ranging from 25 to 90% (Fig. 4), and patients with a diffuse BM [^18^F]FDG uptake with DS ≥ 4 were at higher risk of progression in the univariable analysis [HR (95% CI): 2.46 (1.04–5.83), *P* = 0.040]. However, this was no longer the case in the multivariable analysis when considering transplant in the model [HR (95% CI): 1.29 (0.22–7.52, *P* = 0.779]. This result differs from Deng et al. who showed that baseline BM DS ≥ 4 was independently associated with OS [19].

Similarly to previous studies, the number of FLs at baseline was a prognostic factor, with a high number of FL (*n* > 10; F group 4), reflecting a high tumor burden, being a prognostic factor for OS in our population (Table 3) [12, 15, 33]. However, the wide range of the 95% CI [F group 4: HR (95% CI): 19.10 (1.90–191.95, *P* = 0.010] introduces significant uncertainty into the findings. One of the primary limitations is the small sample size with only 40 patients included, and the low number of observed deaths (13 out of 40; 32.5%) throughout the follow-up period. The Suppl. Table 2. describes the difference of patient characteristics between the IMPeTUs F groups. Tumor burden is a well-known major factor affecting survival in MM patients. MM is an incurable hematological malignancy with variable clinical outcomes, ranging from a few years to more than 10 years, depending on host factors, tumor burden (stage), biology (cytogenetic abnormalities), and depth of response to therapy [34]. In our population, undergoing a transplant was significantly associated with PFS, and the presence of high-risk cytogenetic abnormalities was an independent prognostic factor in term of OS. The association between ISS stage, R-ISS, LDH and serum β2-microglobulin with survival did not reach statistical significance most likely due to the small number of patients included in this study. The number of osteolytic lesions, reflecting bone destruction, was not a significant prognostic factor (Table 3).

In our study, the EMD was not significantly associated with PFS or OS likely due to the low prevalence in our population (*n* = 2/40; 5.0%) [12–14, 35]. Contrary to previous studies, there was no significant association between the presence of PMD and survival [14, 21]. Similarly to the research group of Nantes, who recently assessed the prognostic value of baseline [^18^F]FDG PET biomarkers in NDMM patients included in the prospective multicenter CASSIOPET study, the prognostic value of SUV_max_ using a threshold of 4.2 was not significant in our sample [14, 21]. Previous studies showed an independent prognostic significance of SUV_max_ when using a higher threshold, SUV_max_ ≥ 5.3 and SUV_max_ > 7.1, respectively [13, 36].

Using a SUV4.0 threshold, the present work did not show the prognostic value of the delineated TMTV. This finding contradicts prior research indicating TMTV's significant independent prognostic value for both PFS and OS in NDMM patients. However, it is worth noting that many of these studies employed a lower SUV threshold, typically set at 2.5, for delineating TMTV. [22–26, 28]. Nonetheless, Jamet et al. demonstrated the lower prognostic significance of volume-derived metabolic parameters such as TMTV or TLG compared to BM SUV_max_ [29]. The TMTVs tested were derived from three different segmentation methods: a threshold SUV ≥ 2.5, a fixed threshold at 40% of the SUV_max_, and a K-means clustering algorithm with 2 clusters [29]. The SUV4.0 threshold used to delineate TMTV in the present work was based on three assumptions. First, the choice of SUV4.0 rather than SUV2.5 was based on the work of Zamagni et al. which showed that FL with SUV > 4.2 at baseline independently adversely affected PFS and OS, [12, 37]. Secondly, opting for SUV4.0 instead of the SUV4.2 threshold aimed at facilitating the automatic workflow, drawing on the approach of Barrington et al*.* in lymphoma research, also preventing the selection and delineation of healthy tissues such as the liver (median liver SUV_max_, range: 3.1, 2.5–4.2) [38]. The selected stringent threshold (SUV4.0) surpassed the threshold that would have been determined using the liver SUV_mean_. In our population, a method based on the liver SUV_mean_ plus 1.5 or 2 times the standard deviation would have generated thresholds ranging from 2.1 to 3.4 and 2.2 to 3.6, respectively. An SUV2.1 threshold would have been inappropriate and would have necessitated more manual adjustment, which is crucial to avoid for future implementation in clinical practice. The SUV4.0 threshold was reinforced by the results of Morales-Lozano et al*.* who showed that, among different SUV thresholds tested to delineate TMTV, the second most relevant threshold was SUV > 4 [37]. Thirdly, MM is recognized for displaying spatial heterogeneity, with sub-clones from FL with unfavorable genomic profiles being more prone to therapy resistance [11, 33, 39, 40]. In the post-treatment setting, Zamagni et al*.* demonstrated that persistent FL with SUV_max_ > 4.2 or more recently persistent FL and/or BM [^18^F]FDG uptake with a Deauville-scale score ≥ 4 were independent prognostic biomarkers [12, 32]. In this study, post-treatment [^18^F]FDG PET/CT scans were unavailable. Instead, we focused on evaluating the potential prognostic value of the potentially clinically relevant high-risk tumor burden ([^18^F]FDG avid lesions delineated using the SUV4.0 threshold) based solely on the baseline [^18^F]FDG PET/CT scans.

The absence of prognostic significance of TMTV delineated using the SUV4.0 threshold in our population might be related to the limited number of patients (n = 40), but this threshold might also underestimate tumor burden, that is a well-known prognostic factor (Fig. 2). Deep learning-based tools for automated TMTV delineation might be a faster tool to more accurately delineate [^18^F]FDG-positive tumor burden [41]. Sachpekidis et al. showed that the TMTV obtained using a deep learning–based delineation method in previously untreated MM patients was significantly correlated with BM plasma cell infiltration and was associated with PFS and OS [20]. Nevertheless, the number of delineated VOI using the SUV4.0 threshold, i.e. the number of FL with high [^18^F]FDG uptake with a SUV ≥ 4, was an independent prognostic factor associated with worse PFS.

Post-treatment [^18^F]FDG PET/CT was not available in enough patients to assess the depth of response as an additional biomarker. The present work is mainly limited by the retrospective design, the heterogeneity of first-line treatments, and the small number of included patients (n = 40), which accounts for the low statistical power. With the exception of research groups who investigated TMTV in patients enrolled in the Total Therapy 3A, IFM/DFCI2009, EMN02/HO95 and CASSIOPET trials, the majority of previous studies investigating the prognostic value of IMPeTUs criteria and volumetric parameters at baseline included a limited number of patients [14, 15, 22–25, 28, 29, 37, 40, 42, 43]. The limited number of patients included in the trials poses a challenge for drawing definitive conclusions regarding the prognostic value and clinical utility of baseline [^18^F]FDG PET and TMTV measurements in MM patients.

## Conclusions

Our work revealed that a high number of FL (*n* > 10; IMPeTUs F group 4), indicative of [^18^F]FDG-avid tumor burden, emerged as a prognostic factor for OS. The prognostic value of the TMTV defined using a SUV4.0 threshold was not statistically significant; nevertheless, the count of delineated [^18^F]FDG-avid outlined lesions (VOI) using a SUV4.0 threshold was an independent prognostic factor for PFS.

### Supplementary Information

Additional file 1.

## Data Availability

The data presented in this study are available on request from the corresponding author (smarchiori@chuliege.be).

## Abbreviations

[^18^F]FDG: 2-[^18^F]fluoro-2-deoxy-D-glucose
ASCT: Autologous stem-cell transplantation
BM: Bone marrow
BMPC: Clonal bone marrow plasma cell
CI: Confidence interval
Cox-ph: Cox proportional hazards models
DS: Deauville-scale
EMD: Extramedullary disease
FL: Focal lesions
FLC: Free light chain
HR: Hazard ratio
ICC: Intra-class correlation
IMPeTUs: Italian myeloma criteria for PET Use
LDH: Lactate deshydrogenase
NDMM: Newly diagnosed multiple myeloma
OS: Overall survival
PET/CT: Positron emission tomography combined with low-dose computed
PFS: Progression free survival
PMD: Paramedullary disease
R-ISS: Revised International Staging System
SUV: Standard uptake value
TLG: Total lesion glycolysis
TMTV: Total metabolic tumor volume
VOI: Volume of interest
