# Supplementary material for: Prognostic value of visual IMPeTUs criteria and metabolic tumor burden at baseline [18F]FDG PET/CT in patients with newly diagnosed multiple myeloma
Source: EJNMMI Res. 2024 May 28;14:51. doi: 10.1186/s13550-024-01113-6 (PMC11133264; doi:10.1186/s13550-024-01113-6)
Supplement: Supplementary file 1 — Additional file 1. [file 13550_2024_1113_MOESM1_ESM.docx]

**SUPPLEMENTAL DATA**

**[^18^F]FDG PET/CT acquisition parameters**

[^18^F]FDG PET/CT scans were acquired using 2 cross-calibrated EARL-accredited PET/CT systems, a GEMINI TF Big Bore and a GEMINI TF 16 (Philips) after intravenous injection of [^18^F]FDG (median uptake time, 64 min and range, 51–108 min; mean injected activity, 234 MBq, depending linearly on the patient’s weight). The patients fasted for at least 6 h before the injection, and the median glycemia was 96 mg/dL (range, 72-138 mg/dL).

A low-dose CT scan (slice thickness, 3 mm; tube voltage, 120 kV; and tube current–time product, 50–80 mAs, depending on the patient’s weight) was performed without injection of intravenous contrast agent, followed by a PET emission scan with a time per bed position (pbp) depending on the patient's body mass index (BMI ≤ 25: 1 min pbp; BMI ≥ 26 and ≤ 32: 1 min 30 sec pbp; BMI ≥ 33: 2 min pbp), with 50% overlap The field of view extended from vertex to knees in the majority of patients (*n* = 29/40; 72,5%), from skull base to mid-thigh in 7 patients (17.5%), from vertex to mid-thigh in 3 patients (7.5%) and from skull base to knees in 1 patient (2.5%). All images were acquired and reconstructed according to the EARL (European Association of Nuclear Medicine Research Ltd.) guidelines for both PET/CT systems. Images were reconstructed with standard 4 x 4 x 4 mm voxels (slice thickness, 4 mm) using an iterative list-mode algorithm (blob ordered-subset time-of-flight), and corrections for attenuation, dead time, random events, and scatter events were applied without post-reconstruction smoothing.

**SUPPLEMENTAL TABLE 1.**

[^18^F]FDG PET/CT results

| [^18^F]FDG PET/CT parameter | Value  *n* = 40 (100%) | Specified if missing, n (%) |
| --- | --- | --- |
| **Negative PET/CT** | 2 (5) |  |
| **Diffuse bone marrow Deauville scale score** |  |  |
| BM DS 1 | 1 (2.5) |  |
| BM DS 2 | 19 (47.5) |  |
| BM DS 3 | 12 (30.0) |  |
| BM DS 4 | 7 (17.5) |  |
| BM DS 5 | 1 (2.5) |  |
| **Location of FL &/or osteolytic lesions** |  |  |
| Presence of skull FL^†^ | 18 (45.0) | 7 (17.5)^††^ |
| Presence of spinal FL^†^ | 27 (67.5) |  |
| Presence of extraspinal FL^†^ | 30 (75.0) |  |
| **Number of bone FL by IMPeTUs group** |  |  |
| F group 1 (no bone FL) | 11 (27.5) |  |
| F group 2 | 15 (37.5) |  |
| F group 3 | 6 (15.0) |  |
| F group 4 | 8 (20.0) |  |
| **Number of bone FL in PET images** |  |  |
| ≤ 3 | 26 (65.0) |  |
| > 3 | 14 (35.0) |  |
| **Hottest bone FL Deauville scale score** |  | 11 (27.5) |
| FL DS 2 | 3 (7.5) |  |
| FL DS 3 | 7 (17.5) |  |
| FL DS 4 | 6 (15.0) |  |
| FL DS 5 | 13 (32.5) |  |
| **Number of osteolytic FL in CT images** |  |  |
| L group 1 (no osteolytic FL) | 4 (10.0) |  |
| L group 2 | 9 (22.5) |  |
| L group 3 | 10 (25.0) |  |
| L group 4 | 17 (42.5) |  |
| **Presence of at least one fracture on CT images** | 20 (50.0) |  |
| **Presence of PMD** | 12 (30.0) |  |
| **PMD Deauville scale score** |  |  |
| PMD DS 2 | 2 (5.0) | 28 (70.0) |
| PMD DS 3 | 1 (2.5) |  |
| PMD DS 4 | 2 (5.0) |  |
| PMD DS 5 | 7 (17.5) |  |
| **Presence of at least one EMD** | 2 (5.0) |  |
| **EMD Deauville scale score:** |  |  |
| EMD DS 3 | 1 (50.0) | 38 (95.0) |
| EMD DS 4 | 1 (50.0) |  |
| **Median (P25-P75) TMTV** | 0.225 (0-15.3) ml | 20 (50.0)^†††^ |
| **Median (P25-P75) TLG** | 1.03 (0-71.4) g | 20 (50.0)^†††^ |
| **Median (P25-P75) SUV_max_** | 4.64 (2.23-6.78) | 8 (20) |
| **Median (P25-P75) SUV_mean_** | 2.21 (0-4.72) | 20 (50.0)^†††^ |
| **Median (range) number of VOI per patient** | 0.5 (0.0-5.25) |  |
| **Presence of a diffuse pattern in CT images** | 18 (45.0) |  |

^†^ Presence of FL based on CT and/or PET

^††^Absence of skull FL but the skull vault was not included in the field of view

^†††^ No delineated volume (no FL and/or SUV_max_ < 4)

Abbreviations: BM, diffuse bone marrow; DS, Deauville scale; EMD, extramedullary disease; FL, focal lesion; FL DS, hottest bone FL Deauville scale score; PMD, paramedullary disease; SUV_max_, maximum standardized uptake value; SUV_mean_, mean standardized uptake value; TMTV, total metabolic tumor volume; TLG, total lesion glycolysis; VOI, volume of interest.

**SUPPLEMENTAL TABLE 2.**

Description and comparison of patient characteristics between the IMPeTUs

F groups (1: no lesion; 2: n = 1-3 FL; 3: n = 4-10 FL and 4: n > 10 FL)

| **Variables** | **F groups** | | | |  |
| --- | --- | --- | --- | --- | --- |
|  | F1 (N=11) | F2 (N=15) | F3 (N=6) | F4 (N=8) | p-value |
| **Age (years)** |  |  |  |  | 0.815 |
| Median (Q1-Q3) | 63.2  (56.8-67.1) | 62.9  (54.7-69.7) | 60.2  (53.3-66.1) | 65.0  (61.6-68.3) |  |
| **Sexe** |  |  |  |  | 0.413 |
| Female | 4 (36.4%) | 9 (60.0%) | 3 (50.0%) | 2 (25.0%) |  |
| Male | 7 (63.6%) | 6 (40.0%) | 3 (50.0%) | 6 (75.0%) |  |
| **Hemoglobin (g/L)** |  |  |  |  | 0.341 |
| Median (Q1-Q3) | 11.7  (9.53-12.2) | 12.4  (10.6-14.4) | 10.4  (8.95-11.4) | 11.4  (10.9-12.5) |  |
| Missing | 1 (9.1%) | 1 (6.7%) | 0 (0%) | 0 (0%) |  |
| **ISS stage** |  |  |  |  | 0.295 |
| I | 6 (60.0%) | 10 (76.9%) | 2 (33.3%) | 3 (37.5%) |  |
| II | 2 (20.0%) | 3 (23.1%) | 2 (33.3%) | 3 (37.5%) |  |
| III | 2 (20.0%) | 0 (0%) | 2 (33.3%) | 2 (25.0%) |  |
| Missing | 1 (9.1%) | 2 (13.3%) | 0 (0%) | 0 (0%) |  |
| **R-ISS stage** |  |  |  |  | 0.113 |
| I | 2 (20.0%) | 7 (53.8%) | 0 (0%) | 2 (25.0%) |  |
| II | 7 (70.0%) | 6 (46.2%) | 4 (66.7%) | 4 (50.0%) |  |
| III | 1 (10.0%) | 0 (0%) | 2 (33.3%) | 2 (25.0%) |  |
| Missing | 1 (9.1%) | 2 (13.3%) | 0 (0%) | 0 (0%) |  |
| **Serum β2-microglobulin (mg/L)** |  |  |  |  | 0.240 |
| Median (Q1-Q3) | 3.57  (2.67-5.45) | 2.42  (2.16-3.45) | 3.26  (2.26-10.4) | 3.78  (2.86-4.33) |  |
| Missing | 3 (27.3%) | 4 (26.7%) | 0 (0%) | 0 (0%) |  |
| **β2-microglobulin** |  |  |  |  | 0.401 |
| [0,3.5) | 4 (50.0%) | 8 (72.7%) | 3 (50.0%) | 3 (37.5%) |  |
| [3.5,5.4] | 2 (25.0%) | 3 (27.3%) | 1 (16.7%) | 4 (50.0%) |  |
| (5.4+) | 2 (25.0%) | 0 (0%) | 2 (33.3%) | 1 (12.5%) |  |
| Missing | 3 (27.3%) | 4 (26.7%) | 0 (0%) | 0 (0%) |  |
| **Serum Albumin (g/L)** |  |  |  |  | 0.674 |
| Median (Q1-Q3) | 39.1  (37.1-46.1) | 44.0  (41.6-46.0) | 38.5  (35.1-43.5) | 40.8  (38.2-42.8) |  |
| Missing | 1 (9.1%) | 2 (13.3%) | 0 (0%) | 0 (0%) |  |
| **Albumin** |  |  |  |  | 0.742 |
| [0,35) | 1 (10.0%) | 3 (23.1%) | 2 (33.3%) | 2 (25.0%) |  |
| [35+) | 9 (90.0%) | 10 (76.9%) | 4 (66.7%) | 6 (75.0%) |  |
| Missing | 1 (9.1%) | 2 (13.3%) | 0 (0%) | 0 (0%) |  |
| **LDH (U/L)** |  |  |  |  | 0.011 |
| Median (Q1-Q3) | 192  (166-222) | 175  (156-199) | 239  (236-278) | 196  (172-216) |  |
| Missing | 1 (9.1%) | 2 (13.3%) | 0 (0%) | 0 (0%) |  |
| **LDH > the upper limit of normal (220 U/L)** |  |  |  |  | 0.095 |
| Normal | 7 (63.6%) | 10 (76.9%) | 1 (16.7%) | 6 (75.0%) |  |
| High | 4 (36.4%) | 3 (23.1%) | 5 (83.3%) | 2 (25.0%) |  |
| Missing | 0 (0%) | 2 (13.3%) | 0 (0%) | 0 (0%) |  |
| **Serum M protein IgG/IgA (g/L)** |  |  |  |  | 0.871 |
| Median (Q1-Q3) | 23.9  (15.6-36.8) | 19.8  (12.2-31.7) | 27.7  (20.3-40.4) | 28.1  (14.4-36.6) |  |
| Missing | 3 (27.3%) | 6 (40.0%) | 2 (33.3%) | 1 (12.5%) |  |
| **Serum free light chain (kappa/lambda) ratio** |  |  |  |  | 0.596 |
| Median (Q1-Q3) | 1.60  (0.01-148) | 5.95  (0.06-93.30) | 0.900  (0.06-35.70) | 8.87  (0.18-47.90) |  |
| Missing | 1 (9.1%) | 3 (20.0%) | 1 (16.7%) | 0 (0%) |  |
| **Serum creatinine (mg/dL)** |  |  |  |  | 0.031 |
| Median (Q1-Q3) | 0.970 (0.870-1.34) | 0.795  (0.630-1.03) | 1.06  (0.785-3.69) | 0.910  (0.763-1.12) |  |
| Missing | 2 (18.2%) | 1 (6.7%) | 0 (0%) | 0 (0%) |  |
| **Calcium (mmol/L)** |  |  |  |  | 0.504 |
| Median (Q1-Q3) | 2.44  (2.32-2.56) | 2.37  (2.35-2.45) | 2.42  (2.38-2.47) | 2.52  (2.47-2.57) |  |
| Missing | 1 (9.1%) | 2 (13.3%) | 0 (0%) | 0 (0%) |  |
| **BMPC (%)** |  |  |  |  | 0.466 |
| Median (Q1-Q3) | 43.0  (17.0-57.0) | 20.0  (11.0-35.0) | 36.0  (27.5-69.3) | 35.0  (28.8-52.0) |  |
| Missing | 0 (0%) | 2 (13.3%) | 0 (0%) | 0 (0%) |  |
| **BMPC** |  |  |  |  | 0.678 |
| [0,10) | 0 (0%) | 3 (23.1%) | 0 (0%) | 0 (0%) |  |
| [10,59] | 8 (72.7%) | 7 (53.8%) | 4 (66.7%) | 6 (75.0%) |  |
| (59+) | 3 (27.3%) | 3 (23.1%) | 2 (33.3%) | 2 (25.0%) |  |
| Missing | 0 (0%) | 2 (13.3%) | 0 (0%) | 0 (0%) |  |
| **Kappa Free (mg/L)** |  |  |  |  | 0.596 |
| Median (Q1-Q3) | 14.5  (5.73-155) | 15.9  (9.04-280) | 27.5  (9.69-248) | 42.2  (10.3-141) |  |
| Missing | 1 (9.1%) | 3 (20.0%) | 1 (16.7%) | 0 (0%) |  |
| **Lambda Free (mg/L)** |  |  |  |  | 0.528 |
| Median (Q1-Q3) | 83.5  (3.85-567) | 11.8  (5.21-460) | 27.9  (10.7-499) | 21.7  (3.53-61.1) |  |
| Missing | 1 (9.1%) | 3 (20.0%) | 1 (16.7%) | 0 (0%) |  |
| **Induction** |  |  |  |  | 0.827 |
| DRd | 1 (9.1%) | 1 (6.7%) | 0 (0%) | 0 (0%) |  |
| Rmed | 1 (9.1%) | 0 (0%) | 0 (0%) | 0 (0%) |  |
| Vd | 1 (9.1%) | 0 (0%) | 0 (0%) | 0 (0%) |  |
| VRd | 1 (9.1%) | 1 (6.7%) | 0 (0%) | 0 (0%) |  |
| VTd | 7 (63.6%) | 7 (46.7%) | 5 (83.3%) | 6 (75.0%) |  |
| Rd | 0 (0%) | 1 (6.7%) | 1 (16.7%) | 0 (0%) |  |
| RdE | 0 (0%) | 1 (6.7%) | 0 (0%) | 0 (0%) |  |
| VCd | 0 (0%) | 3 (20.0%) | 0 (0%) | 0 (0%) |  |
| VMp | 0 (0%) | 1 (6.7%) | 0 (0%) | 1 (12.5%) |  |
| DVd | 0 (0%) | 0 (0%) | 0 (0%) | 1 (12.5%) |  |
| **Induction+Transplant** |  |  |  |  | 0.472 |
| OtherInduction+NoTransplant | 4 (36.4%) | 6 (40.0%) | 0 (0%) | 2 (25.0%) |  |
| VTd+NoTransplant | 1 (9.1%) | 1 (6.7%) | 1 (16.7%) | 0 (0%) |  |
| VTd+Transplant | 6 (54.5%) | 6 (40.0%) | 4 (66.7%) | 6 (75.0%) |  |
| OtherInduction+Transplant | 0 (0%) | 2 (13.3%) | 1 (16.7%) | 0 (0%) |  |
| **Transplant** |  |  |  |  | 0.549 |
| No | 5 (45.5%) | 7 (46.7%) | 1 (16.7%) | 2 (25.0%) |  |
| Yes | 6 (54.5%) | 8 (53.3%) | 5 (83.3%) | 6 (75.0%) |  |
| P-value: Fisher’s exact test for qualitative variables and One-Way Anova for quantitative variables. | | | | | |

Abbreviation: BMPC, Bone marrow plasma cell percentage; DRd, Daratumumab-Lenalidomide-Dexamethasone; DVd, Daratumumab-Bortezomib-Dexamethasone; FL, Focal lesion; ISS, International Staging System; LDH, lactate deshydrogénase; R-ISS, Revised-ISS; Rd, Lenalidomide-Dexamethasone; RdE, Lenalidomide-Dexamethasone-Elotuzumab; Rmed, Lenalidomide-Medroxyprogesterone; Vd, Bortezomib-Dexamethasone; VCd, Bortezomib-Cyclophosphamide-Dexamethasone; VMp, Bortezomib-Melphalan-Prednisone; VRd, Bortezomib-Lenalidomide-Dexamethasone; VTd, Bortezomib-Thalidomide-Dexamethasone.
